# The Prognostic Value of Tumor‐Associated Neutrophils in Colorectal Cancer: A Systematic Review and Meta‐Analysis

**DOI:** 10.1002/cam4.70614

**Published:** 2025-02-27

**Authors:** Mengyuan Jiang, Rui Zhang, Min Huang, Jing Yang, Qianqian Liu, Ziru Zhao, Ya Ma, Hongfan Zhao, Min Zhang

**Affiliations:** ^1^ Department of Pathology Taicang Loujiang New City Hospital Taicang Jiangsu China; ^2^ Department of Pathology Gansu Provincial Hospital Lanzhou Gansu China; ^3^ The First School of Clinical Medicine Gansu University of Traditional Chinese Medicine Lanzhou Gansu China; ^4^ Department of Pathology The 940th Hospital of Joint Logistics Support Force of Chinese People´s Liberation Army Lanzhou Gansu China; ^5^ Department of Pathology Gansu Provincial Cancer Hospital Lanzhou Gansu China; ^6^ Department of Pathology Chengdu First People's Hospital Chengdu Sichuan China; ^7^ The First School of Clinical Medicine Lanzhou University Lanzhou Gansu China; ^8^ Clinical Research Centre, Department of Science and Technology Sichuan Provincial People's Hospital, University of Electronic Science and Technology of China Chengdu Sichuan China

**Keywords:** colorectal cancer, meta‐analysis, prognosis, tumor‐associated neutrophils

## Abstract

**Background:**

Tumor‐associated neutrophils (TANs) are important components of the colorectal cancer (CRC) microenvironment. However, their role in CRC remains controversial. This study aimed to assess the prognostic value of TANs in patients with CRC.

**Methods:**

We searched the PubMed, EMBASE, and Cochrane Library databases for eligible studies published until January 9, 2023. The pooled hazard ratios (HRs) and odds ratios (ORs) with their 95% confidence intervals (95% CI) were calculated with a random‐effects model to assess survival outcomes and clinicopathological features. Subgroup analyses were further conducted to identify potential sources of heterogeneity. Funnel plots and Egger's test were used to measure publication bias.

**Results:**

A total of 19 studies with 7721 patients were included in this meta‐analysis. The pooled analysis indicated that high peritumoral TAN infiltration in CRC tissue was significantly associated with favorable cancer‐specific survival (HR = 0.57; 95% CI: 0.38–0.86; *p* = 0.007), but not with overall survival or disease‐free survival. No association between high intratumoral or unclear compartment TAN infiltration and CRC prognosis was found. Subgroup analyses showed that the association between TANs and the prognosis of CRC patients differed according to antibody types, tumor stage, quantitative methods, and follow‐up time. High intratumoral TAN infiltration was significantly associated with histology type, whereas high TAN infiltration in an unclear compartment was significantly associated with gender, tumor location, and the primary tumor site.

**Conclusions:**

High TAN infiltration, especially in the peritumoral compartment, could be a potential prognostic marker in CRC. More high‐quality studies are required to explore its specific prognostic value in CRC.

## 1 Introduction

Colorectal cancer (CRC) is the third most common cancer and is one of the leading causes of cancer‐related death worldwide [1]. The incidence and mortality of CRC in many developing countries or less‐developed regions are increasing [2]. CRC treatment has made a great progress with improvements in early screening and modern technology. However, CRC is a highly heterogeneous disease with abundant histological subtypes, pathological markers, inflammatory infiltration, and molecular subtypes, presenting great challenges for personalized clinical diagnosis and treatment [3]. The Tumor, Node, and Metastasis (TNM) classification system is currently the most commonly used prognostic tool for tumors. However, survival outcomes vary widely among CRC patients even with the same TNM stage. Therefore, accurate prognostic markers for CRC are urgently needed to indicate disease progression and prognosis and to guide precision medicine.

Inflammation is a critical component of the tumor microenvironment (TME) [4], which is a key player in tumor development and progression [5, 6]. Neutrophils, the most abundant leukocytes, are the first line of defense against invading pathogens [7]. Tumor‐associated neutrophils (TANs) are neutrophils that accumulate at tumor sites under the action of chemokines and are major players in the TME [8]. Past studies considered neutrophils to be present for a short‐term and ignored their role in tumors. However, recent studies confirmed the role of neutrophils in tumor progression [9, 10]. TANs appear to play a dual role in tumor progression. TANs play an anti‐tumor role by regulating their cytotoxic effect on tumor cells and inhibiting metastasis [11, 12]. However, they also play a pro‐cancer role by promoting tumor angiogenesis [13, 14], immunosuppression [15, 16, 17], and tumor metastasis [18, 19]. In most solid tumors, TANs suggest a poor prognosis [20], and this needs more investigation.

The prognostic value of TANs in CRC has been extensively studied in recent years [21, 22]. However, the correlation between TANs and CRC prognosis is controversial. Therefore, we performed a systematic review and meta‐analysis to explore the roles and value of TANs in the prognosis and clinicopathological features of CRC patients and to provide clues for CRC immunotherapy.

## 2 Methods

The protocol for this systematic review and meta‐analysis was registered with the International Prospective Register of Systematic Reviews (PROSPERO) (identification code CRD42022329368).

### 2.1 Search Strategy

A systematic search was conducted for this systematic review and meta‐analysis through PubMed, EMBASE, and the Cochrane Library databases for eligible studies reported until January 9, 2023, with the following terms: “neutrophils” AND (“colorectal cancer” OR “colorectal neoplasm” OR “colorectal carcinoma” OR “colorectal tumor”) AND “prognosis.” The search for these terms was restricted to the title/abstract without other restrictions. The detailed search strategy for each database is described in Table S1. These database searches were complemented with potentially relevant articles by searching the reference lists of all relevant research articles and screening reviews.

### 2.2 Study Selection

Primary studies had to meet the following criteria for inclusion in this article: (1) analyzed the expression of TANs either from surgery resection or biopsy for CRC, (2) the patient underwent no prior radiotherapy and/or chemotherapy, (3) the study reported a correlation between TANs and the prognosis of CRC patients with sufficient data available to acquire the hazard ratios (HRs) and their 95% confidence intervals (CIs), (4) TANs were divided into high and low values using a certain cut‐off criterion, and (5) studies published as full papers in English or Chinese. Accordingly, studies were excluded if the following conditions were met: (1) studies with fewer than 30 participants; (2) duplicate articles, case series, letters, reviews, abstracts, commentaries, editorials, and meeting reports; (3) studies without enough data on prognoses; and (4) studies on metastatic CRC. We adopted the most comprehensive or largest sample size reports for studies that included duplicated databases or tissue microarrays.

The prognostic outcomes were overall survival (OS), disease‐free survival (DFS), recurrence‐free survival (RFS), cancer‐specific survival (CSS), and disease‐specific survival (DSS). For convenience, we unified DFS and RFS into DFS, and CSS and DSS into CSS.

### 2.3 Data Extraction and Quality Assessment

Data extraction was independently performed by two reviewers (Mengyuan Jiang and Rui Zhang), and any disputes were settled by discussion with a third author (Min Huang). The following data and information were extracted: (1) publication data, including the first author and year of publication; (2) demographic data, including the country of the specimen, sample size, median/mean age, sex ratio of the patients, and follow‐up duration; (3) tumor information on the type of cancer, tumor site, and tumor stage (4) experimental data involving TAN location, method of testing TAN expression, antibodies tested, definition of high or low TAN values and the cutoff value; and (5) statistical data, including HRs with 95% CIs and all adjusted factors used for multivariate analysis. We grouped TAN locations into the intratumoral (IT) compartment, peritumoral (PT) compartment, and unclear compartment to facilitate meta‐analysis classification and analysis. Tumors located in the tumor center, tumor nest, or intratumor region/site were classified into the IT compartment. Tumors located in the tumor invasive margin, tumor front, or peritumoral region/site were unified as the PT compartment. Tumors with both IT and PT compartments or where a specific region was not reported were classified as an unclear compartment.

The quality of the included studies was evaluated using the Quality in Prognosis Studies (QUIPS) Tool [23], which mainly consists of six dimensions: study participation, study attrition, prognostic factor measurement, outcome measurement, study confounding, and statistical analysis and reporting. Each dimension was evaluated as having a low risk of bias, moderate risk of bias, or high risk of bias. Two authors (Mengyuan Jiang and Rui Zhang) independently completed the evaluation, and disagreements were resolved by discussion with a third author (Min Huang).

### 2.4 Statistical Analysis

HRs and associated 95% CIs were calculated to investigate the relationship between TAN expression and the prognosis of CRC patients. If the HRs and CIs were not directly available, they were extracted from Kaplan–Meier curves using Engauge Digitizer version 10.6 [24] and estimated using the method reported by Parmar et al. [25]. The odds ratios (ORs) and associated 95% CIs were calculated to assess the association between TAN expression and the clinicopathological features of CRC patients. Heterogeneity was evaluated using Cochran's *Q* statistic and *I* ^2^ tests. A random‐effects model (the DerSimonian‐Laird method) was chosen to calculate the HRs.

Subgroup analyses were performed to identify the potential sources of heterogeneity and assess the impact of various variables on the outcomes. We analyzed several subgroups, including region (Asia or non‐Asia), immune markers (CD66b, CD15, or none), type of analysis (automatic computerized quantification, semiquantitative methods, stereology, or gene analysis), follow‐up time (less than 10 years or more than 10 years), and tumor stage (Stage I–III or Stage I–IV).

Funnel plots and Egger's test were used to measure publication bias. Sensitivity analysis was conducted to evaluate the stability of the results in all articles. R statistical software (version 4.1.3) and STATA software (version 16.0) were used to conduct statistical analyses. A two‐tailed *p*‐value of ≤ 0.05 was considered statistically significant.

## 3 Results

### 3.1 Study Selection and Baseline Characteristics

**FIGURE 1:**
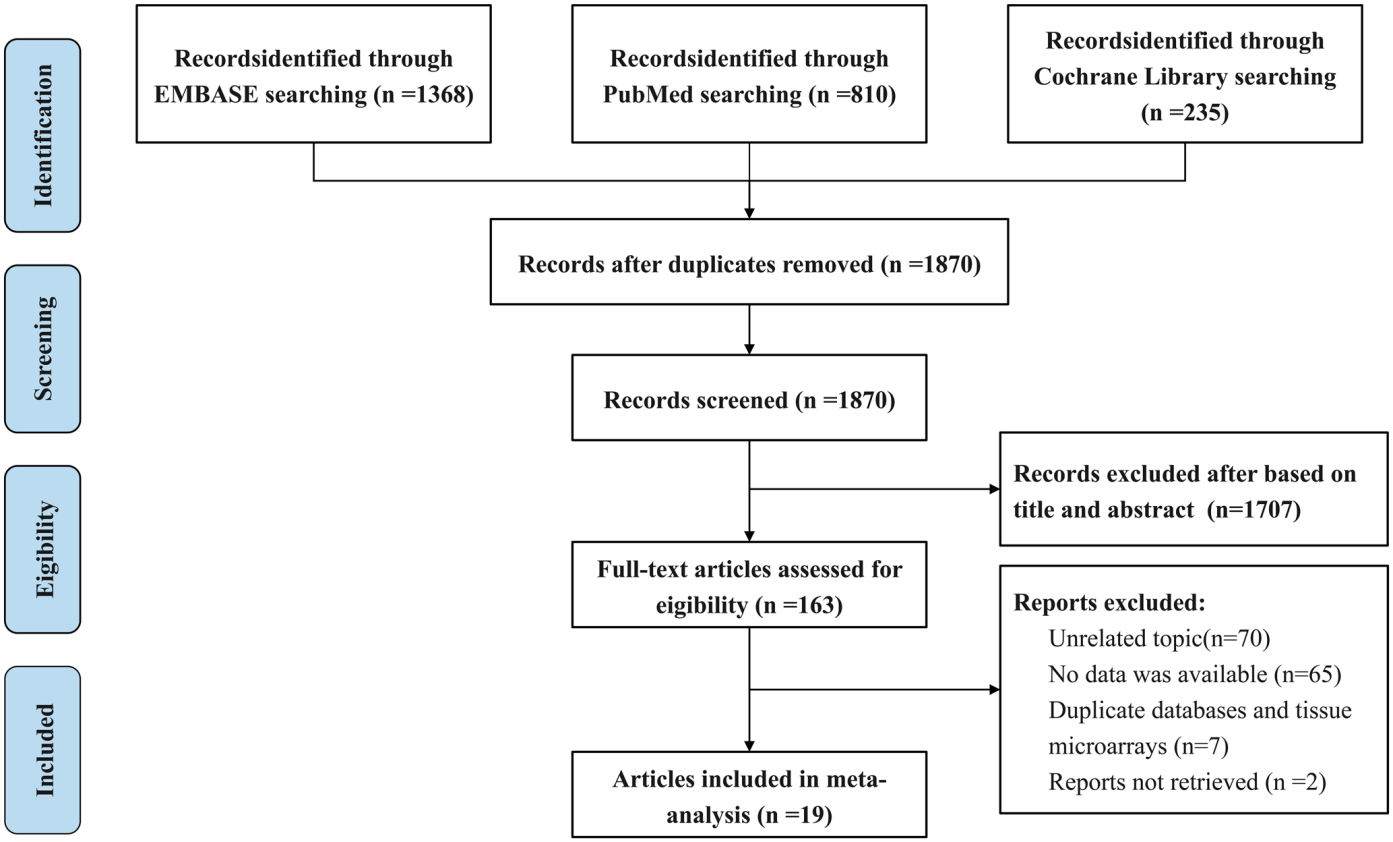
Flow diagram of the study selection process.

Initially, we retrieved 2413 studies from the systematic search strategies. A total of 1870 records were identified after removing duplicates. After screening titles and abstracts, the full texts of the remaining 163 papers were carefully reviewed to determine whether they met the inclusion or exclusion criteria, and 144 of them were excluded. Finally, 19 full‐text articles published between 2005 and 2022 were included in the meta‐analysis [21, 22, 26, 27, 28, 29, 30, 31, 32, 33, 34, 35, 36, 37, 38, 39, 40, 41, 42] (Figure 1).

The main characteristics of the 19 included articles containing 7721 CRC patients are shown in Table 1 and Table S2. These studies were mostly conducted in Asia. The sample sizes ranged from 72 to 1225, and the follow‐up duration ranged from 50 months to 200 months. Of the 19 articles, nine studies distinguished IT and PT TANs, while 10 studies did not specify the location of the TANs. All articles used immunohistochemical staining except for 4 studies that used hematoxylin and eosin (H&E) staining and one study that used gene analysis to detect the expression of TANs. The immune markers used were also different. Eleven studies used CD66b to detect TAN density, CD15 was used in two, and other specific immunomarkers (CD177) were used in one. Eighteen studies described the TNM stage. The quantitative methods used to identify TANs and the cutoff criteria of TANs in these studies were varied. Regarding prognostic outcomes, 12 studies assessed the association between TANs and OS, 10 studies assessed DFS, and five studies assessed CSS.

**TABLE 1:** Characteristics of studies included in this meta‐analysis.

Study	Region	Range of year	No of Patients	Sex (male/female)	Age (years) mean (range) /distribution	Tumor type	Stage	TAN location	Immune marker	Quantitative method	Cut‐off criteria	Outcome
Xu, X. 2021 [29]	China	2009–2012	1021	608/413	60.82 ± 12.51	Colon 606, Rectum415	I–III	IT	CD66b	Stereology	StepMiner algoritjm	DFS, OS
Berry, R. S. 2017 [30]	America	1991–2014	221	107/114	61	CRC	I–IV	Undistinguished	‐(H&E)	Stereology	Median	OS
Galdiero, M. R. 2016 [36]	Italy	1997–2006	128	73/55	≤ 65,66; > 65,62	Colon 99, Rectum 29	I–IV	IT+IM	CD66b	Automatic computerized quantification	Median	DFS, DSS
Galdiero, M. R. 2016 [36]	Italy	1997–2006	178	108/70	< 68,97; ≥ 68,81	Colon 125, Rectum 53	III	IT	CD66b	Automatic computerized quantification	Median	DFS
Ye, L. 2019 [42]	China	2001–2009	359	205/154	≤ 60,177; > 60,182	Colon 215, Rectum 144	I–III	NR	CD66b	Stereology	Median	DFS, OS
Ye, L. 2019 [42]	China	2010–2011	249	165/84	≤ 60,131; > 60,118	Colon 84, Rectum 165	I–III	NR	CD66b	Stereology	Median	DFS, OS
Ye, L. 2019 [42]	China	2010–2011	400	226/174	≤ 60,184; > 60,216	Colon 156, Rectum 244	I–III	NR	CD66b	Stereology	Median	DFS, OS
Zhu, B. 2018 [41]	China	2006–2007	337	208/129	< 60,176; ≥ 60,161	Colon 155, Rectum 182	I–IV	Undistinguished	CD66b	Stereology	Median	DFS, OS
Zhu, B. 2018 [41]	China	2008–2009	245	145/100	< 60,125; ≥ 60,120	Colon 108, Rectum 137	I–IV	Undistinguished	CD66b	Stereology	Median	DFS, OS
Rao, H. L. 2012 [21]	China	2000–2006	229	142/87	57.3	Colon 171, rectum 58	I–IV	Tumor center	CD66b	Stereology	ROC	DSS
Rottmann, B. G. 2021 [28]	USA	2011–2017	348	164/184	64.91 ± 14.75	CRC	I–IV	Tumor epithelium/Tumor cell nests	‐(H&E)	Stereology	A semiquantitative manner	RFS
Klintrup, K. 2005 [31]	Finland	1986–1996	374	179/195	67 ± 13	Colon 237, Rectum 137	I–IV	IT+IM	‐(H&E)	Semiquantitative methods	A four‐degree scale	OS
Governa, V. 2017 [33]	Switzerland	1987–1996	677	356/321	69.5	CRC	I–III	Tumor center	CD66b	Stereology	Regression tree analysis	OS
Richards, C. H. 2012 [22]	UK	1997–2006	130	62/68	≤ 64,41; 65–74,47; > 75,42	CRC	I–III	IM	‐(H&E)	Automatic computerized quantification	Median	CSS
Chen, Y. 2016 [35]	China	2000–2006	300	142/158	< 60,144; ≥ 60,156	CRC	I–IV	NR	CD15	Automatic computerized quantification	X‐tile	OS, DFS
Zhu, Y. 2016 [37]	China	2005–2006	210	126/84	≥ 56,118; < 56 92	CRC	I–IV	IT+IM	CD66b	Stereology	Mean	OS, RFS
Mehrabi, S. F. 2021 [26]	Sverige	−1990	72	33/39	≥ 74,39; < 74,33	CC	I–IV	NR	CD66b	NR	ROC	OS
Lin, Y. 2015 [32]	China	2008.1–2008.11	78	46/32	< 60,32; ≥ 60,46	CRC	I–IV	IT/IM	CD15	Stereology	Median	OS
Wikberg, M. L. 2017 [34]	Sweden	1995–2003	398	213/185	≤ 59,67; 60–69,101; 70–79,145; ≥ 80,85	CC	I–IV	IF	CD66b	Semiquantitative methods	A four‐degree scale	CSS
Xiong, Y. 2018 [38]	—	−2017	1011	NR	NR	CRC	NR	NR	‐(gene analysis)	Gene analysis	NR	DFS
Edin, S. 2019 [39]	Finnish	1998–2003	275	143/132	≤ 59,74; 60–69,73; 70–79 83; ≥ 80,45	Colon 128, Rectum 147	I–IV	Undistinguished	CD66b	Automatic computerized quantification	Median	DSS
Zhou, G. 2018 [27]	China	2008–2010	378	209/169	> 60,214; ≤ 60,164	CRC	I–IV	Undistinguished	CD177	Stereology	Median	OS, DFS
Chengzeng, Y.2022 [40]	Japan	2013–2015	103	63/40	71 (38–94)	Colon 59; Rectum 44	I–III	IM	CD66b	Automatic computerized quantification	ROC	OS, DFS

Abbreviations: CC, colonic cancer; CRC, colorectal cancer; CSS, cancer‐specific survival; DFS, disease‐free survival; DSS, disease‐specific survival; IHC, immunohistochemistry; IT, intratumoral; PT, peritumoral; IM, invasion margin; IF, invasive front; NR, not report; OS, overall survival; RFS, recurrence‐free survival; ROC, receiver operating characteristic curve; TANs, tumor‐associated neutrophils; X‐tile, X‐tile bioinformatics software.

The quality assessment of the included studies showed that eight used unadjusted data, which might lead to confounding bias. Therefore, three articles were assessed as high risk, five as medium risk, and 11 as low risk (Table S3).

### 3.2 TAN Infiltration and OS in CRC

**FIGURE 2:**
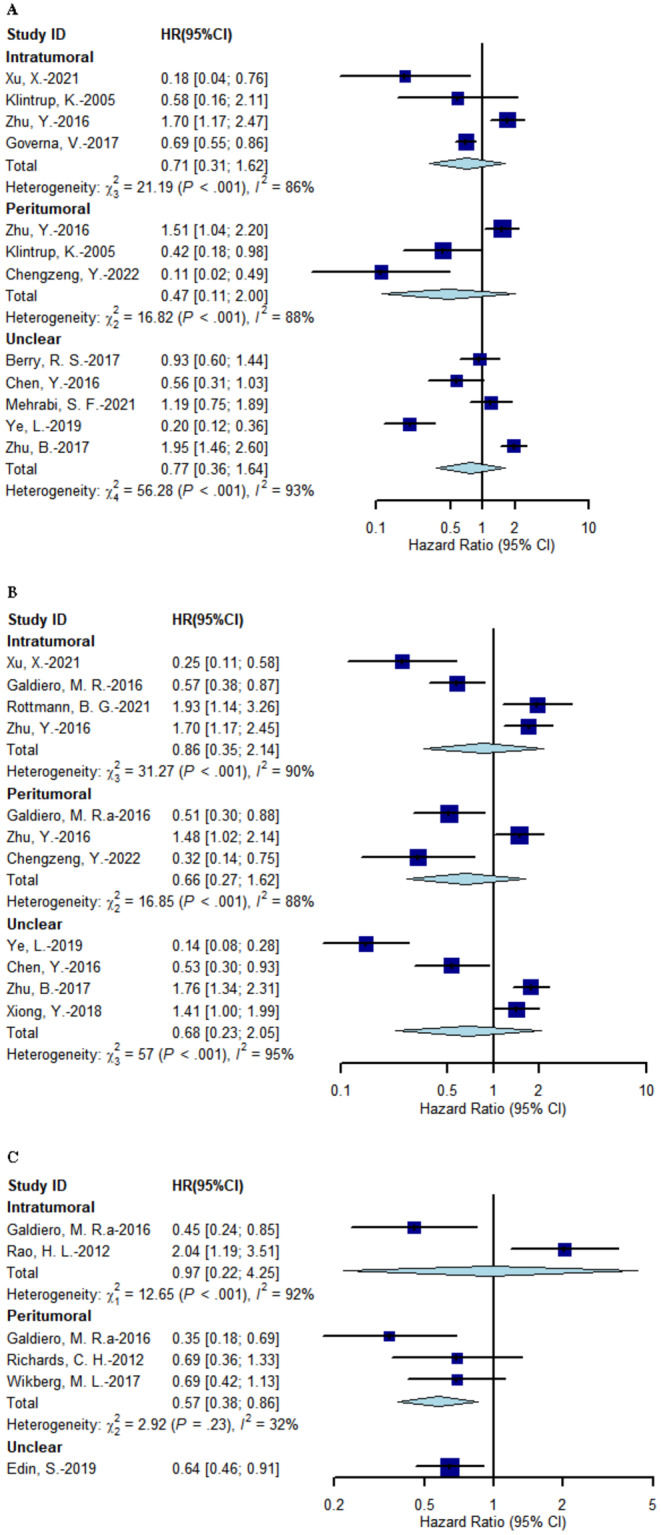
Meta‐analysis for the correlation between TAN expression and overall survival (A), disease‐free survival (B) and cancer‐specific survival (C). HR: Hazard ratio.

Ten studies, including 4646 patients, evaluated the prognostic value of TANs on OS in CRC [26, 29, 30, 31, 33, 35, 37, 40, 41, 42]. No relationship was identified between TANs expression and OS (Figure 2A). No statistical difference in TAN location subgroups was found (*p* = 0.176), but substantial heterogeneity existed within subgroups. Therefore, further subgroup analyses were performed according to IT and unclear compartment TANs rather than PT TANs because those studies were inadequate.

For IT TANs, the results of subgroup analysis showed no significant difference between these subgroups. When stratified by region, high TAN infiltration was associated with favorable OS in non‐Asians (HR = 0.69; 95% CI: 0.55–0.86; *I* ^2^ = 0.0%, *p* = 0.795) but not in Asians (Table 2).

**TABLE 2:** Subgroup analyses for overall survival.

Subgroup	No. of studies	No. of patients	Effect size		Heterogeneity		Test for subgroup differences
			HR (95% CI)	*p*	*I* ^2^ (%)	*p*	*p*
*Intratumoral TANs*							
Region							
Asia	2	1231	0.63 (0.07, 5.44)	0.670	88.6	0.003	0.933
Non‐Asia	2	904	0.69 (0.55, 0.86)	< 0.001	0	0.795	
Antibody types							
CD66b	3	1908	0.71 (0.23, 2.15)	0.540	90.4	< 0.001	0.822
None	1	227	0.58 (0.16, 2.11)	0.408	0	—	
TNM stage							
I–III	2	1698	0.43 (0.13, 1.49)	0.184	69.1	0.072	0.209
I–IV	2	437	1.19 (0.44, 3.21)	0.726	59.3	0.117	
Quantitative methods							
Stereology	3	1908	0.71 (0.23, 2.15)	0.540	90.4	< 0.001	0.566
Semiquantitative methods	1	227	0.58 (0.16, 2.11)	0.408	0	—	
Follow‐up time							
Less than 10 years	2	1248	0.34 (0.11, 1.04)	0.059	26.8	0.242	0.116
More than 10 years	2	887	1.07 (0.44, 2.58)	0.883	93.9	< 0.001	
Overall	4	2135	0.72 (0.31, 1.62)	0.420	85.8	< 0.001	
*Unclear compartment TANs*							
Region							
Asia	3	1890	0.62 (0.17, 2.25)	0.466	96.4	< 0.001	0.441
Non‐Asia	2	284	1.04 (0.76, 1.43)	0.793	0	0.447	
Antibody types							
CD66b	3	1653	0.79 (0.21, 3.00)	0.732	96.0	< 0.001	0.412
CD15	1	300	0.56 (0.31, 1.03)	0.055	0	—	
None	1	221	0.93 (0.60, 1.44)	0.743	0	—	
Stage							
I–III	1	1008	0.21 (0.12, 0.36)	< 0.001	0	—	< 0.001
I–IV	4	1166	1.09 (0.66, 1.81)	0.730	82.8	< 0.001	
Quantitative methods							
Stereology	3	1811	0.73 (0.20, 2.66)	0.635	96.1	< 0.001	0.147
Automatic computerized quantification	1	300	0.56 (0.31, 1.03)	0.055	0	—	
Not report	1	63	1.19 (0.75, 1.89)	0.462	0	—	
Follow‐up time							
Less than 10 years	2	1308	0.34 (0.13, 0.90)	0.030	82.7	0.016	0.012
More than 10 years	3	866	1.33 (0.85, 2.08)	0.218	77.2	0.013	
Overall	5	2174	0.77 (0.36, 1.64)	0.501	92.9	< 0.001	

Abbreviations: CI, confidence interval; HR, hazard ratio; TNM, tumor‐node‐metastasis.

For unclear compartment TANs, the tests for subgroup differences showed significant between‐groups differences within the TNM stage (*p* < 0.001) and follow‐up time subgroups (*p* = 0.012) but not within location, immune markers, or the type of analysis subgroups. The results stratified by TNM stage showed that high TAN infiltration was associated with increased OS in patients with TNM stage I–III (HR = 0.21; 95% CI: 0.12–0.36; *I* ^2^ = 0.0%) but not TNM stage I–IV. Regarding follow‐up time, high TAN infiltration was associated with increased OS in follow‐up times of less than 10 years (HR = 0.34; 95% CI: 0.13–0.90; *I* ^2^ = 82.7%, *p* = 0.016) (Table 2).

One study [32] reporting the relative risk (RR) effect size was not included in the above analysis. The pooled RR was 0.79 (95% CI: 0.35–1.76; *p* = 0.56), which is in the same direction as another study that also labeled neutrophils with CD15 (HR = 0.56; 95% CI: 0.30–1.01; *p* = 0.055) [35]. However, both studies only showed a trend toward higher TANs with better prognosis of CRC patients without actual statistical significance.

### 3.3 TAN Infiltration and DFS in CRC

Nine studies, including 4889 patients, evaluated the prognostic value of TANs on DFS in patients with CRC [28, 29, 35, 36, 37, 38, 40, 41, 42]. No relationship was identified between TAN expression and DFS (Figure 2B). No statistical difference was found in TAN location subgroups (*p* = 0.423), and substantial heterogeneity was found within the subgroups. Therefore, further subgroup analyses were performed according to IT and unclear compartment TANs.

For IT TANs, the test for subgroup differences showed a significant between‐groups difference within the TNM stage subgroup (*p* = 0.006) but not within location, immune markers, type of analysis, or follow‐up time subgroups. The results stratified by TNM stage showed that DFS was significantly increased in patients with TNM stage I–III (HR = 0.25; 95% CI: 0.11–0.58; *I* ^*2*^ = 0.0%) but not in TNM stage I–IV (Table 3).

**TABLE 3:** Subgroup analyses for disease‐free survival.

Subgroup	No. of studies	No. of patients	Effect size		Heterogeneity		Test for subgroup differences
			HR (95% CI)	*p*	*I* ^2^ (%)	*p*	*p*
*Intratumoral TANs*							
Region							
Asia	2	1231	0.68 (0.10,4.39)	0.684	94.2	< 0.001	0.705
Non‐Asia	2	476	1.04 (0.32,3.42)	0.947	92.1	< 0.001	
Antibody types							
CD66b	3	1359	0.66 (0.23,1.90)	0.438	92.0	< 0.001	0.075
None	1	348	1.93 (1.14,3.26)	0.014	0	—	
TNM stage							
I–III	1	1021	0.25 (0.11,0.58)	< 0.001	0	—	0.006
I–IV	3	686	1.23 (0.58,2.61)	0.596	89.2	< 0.001	
Quantitative methods							
Stereology	3	1579	0.98 (0.28,3.40)	0.973	89.5	< 0.001	0.425
Automatic computerized quantification	1	128	0.57 (0.38,0.88)	0.009	0	—	
Follow‐up time							
Less than 10 years	3	1497	0.67 (0.22,2.10)	0.496	90.4	< 0.001	0.130
More than 10 years	1	210	1.70 (1.17,2.45)	0.005	0	—	
Overall	4	1707	0.86 (0.35,2.14)	0.749	90.4	< 0.001	
*Unclear compartment TANs*							
Region							
Aisa	3	1890	0.53 (0.13,2.17)	0.374	96.4	< 0.001	0.184
Non‐Asia	1	1011	1.41 (1.00,1.99)	0.049	0	—	
Antibody types							
CD66b	2	1590	0.51 (0.04,5.94)	0.594	97.9	< 0.001	0.012
CD15	1	300	0.53 (0.30,0.93)	0.028	0	—	
None	1	1011	1.41 (1.00,1.99)	0.049	0	—	
TNM stage							
I–III	1	1008	0.14 (0.08,0.28)	< 0.001	0	—	< 0.001
I–IV	3	1893	1.14 (1.00,1.99)	0.049	85.8	< 0.001	
Quantitative methods							
Stereology	2	1590	0.51 (0.04,5.94)	0.594	97.9	< 0.001	0.012
Automatic computerized quantification	1	300	0.53 (0.30,0.93)	0.028	0	—	
Gene analysis	1	1011	1.41 (1.00,1.99)	0.049	0	—	
Follow‐up time							
Less than 10 years	2	1308	0.28 (0.08,1.00)	0.050	88.5	0.003	0.008
More than 10 years	2	1593	1.62 (1.31,2.00)	< 0.001	0	0.324	
Overall	4	2901	0.68 (0.23,2.05)	0.492	94.7	< 0.001	

Abbreviations: CI, confidence interval; HR, hazard ratio; TNM, tumor‐node‐metastasis.

For unclear compartment TANs, the tests for subgroup differences showed that there were significant between‐groups differences within antibody type (*p* = 0.012), tumor stage (*p* < 0.001), quantitative methods (*p* = 0.012), and follow‐up time subgroups (*p* = 0.008). The results stratified by TNM stage showed that DFS was significantly increased in patients with TNM stage I–III (HR = 0.14; 95% CI: 0.08–0.28; *I* ^2^ = 0.0%) but not TNM stage I–IV. When grouped by follow‐up time, DFS was increased in patients with follow‐up times of less than 10 years (HR = 0.28; 95% CI: 0.08–1.00; *I* ^2^ = 88.5%, *p* = 0.003) but not in follow‐up times of more than 10 years (Table 3). It was difficult to accurately evaluate the subgroup analysis of antibody types and quantitative methods due to the few included studies.

### 3.4 TAN Infiltration and CSS in CRC

Five studies, including 1160 patients, evaluated the prognostic value of TANs on CSS in CRC [21, 22, 34, 36, 39]. Patients with high TAN infiltration in the PT compartment had better CSS (HR = 0.57; 95% CI: 0.38–0.86; *I* ^2^ = 31.6%) but not those with IT or unclear compartment infiltration (Figure 2C). No subgroup difference between TAN locations was detected (*p* = 0.061), and substantial heterogeneity within the subgroups was found. Other subgroups were not analyzed because of the few included studies.

### 3.5 TANs With Specific Markers and CRC Prognosis

Many studies have quantified partially labeled neutrophil immunomarkers to improve prognostication, which made it difficult to assess the actual prognostic value of neutrophils. CD177, a specific neutrophil activation marker, only partially represents neutrophils. One study found that high CD177^+^ TAN infiltration was significantly related to increased OS (HR = 0.58; 95% CI: 0.39–0.87, *p* = 0.009) and DFS (HR = 0.51; 95% CI: 0.32–0.80, *p* = 0.004) [27].

### 3.6 TAN Infiltration and Clinicopathological Features in CRC

Nine studies, including 3888 patients, evaluated the relationship between TAN expression and the clinicopathological features of CRC patients [21, 28, 29, 30, 33, 34, 39, 41, 42] (Table 4). For IT compartment TANs, the pooled results showed that high TAN infiltration was significantly associated with the mucinous histology type (OR = 1.52; 95% CI: 1.03–2.24; *I* ^2^ = 0%, *p* = 0.796). In contrast, high TAN infiltration was not associated with age, gender, tumor location, primary tumor site, differential grade, T stage, TNM stage, lymph node status, or microsatellite instability status. For unclear compartment TANs, the pooled results showed that high TAN infiltration was significantly associated with male sex (OR = 0.73; 95% CI: 0.54–0.97; *I* ^2^ = 43.5%, *p* = 0.150), colon cancer (OR = 1.31; 95% CI: 1.00–1.72; *I* ^2^ = 0%, *p* = 0.543), and left‐sided colon cancer (OR = 0.56; 95% CI: 0.34–0.93; *I* ^2^ = 0%, *p* = 0.661). However, high TAN infiltration was not associated with age, differential grade, T stage, TNM stage, lymph node status, serum carcinoembryonic antigen levels, or serum carbohydrate antigen 199 levels.

**TABLE 4:** The relationship between TANs and clinicopathological characteristics.

Clinicopathological features	No. of studies	No. of patients	Pooled OR (95% CI)	*p*	Heterogeneity	
					*I* ^2^ (%)	*p*
Intratumoral TANs						
Age (older vs. younger)	2	650	0.85 (0.59,1.22)	0.372	0	0.377
Gender (male vs. female)	5	2696	0.95 (0.78,1.16)	0.627	0	0.435
Tumor location (colon vs. rectum)	3	1668	1.05 (0.58,1.89)	0.877	78.8	0.009
Primary tumor site (left colon vs. right colon)	3	1279	0.69 (0.30,1.56)	0.369	85.7	< 0.001
Differential grade(G3 vs. G1‐2)	5	2674	1.17 (0.72,1.90)	0.536	55.3	0.062
Histology type (mucinous vs. non‐mucinous)	2	1093	1.52 (1.03,2.24)	0.035	0	0.796
T stage (T3‐4 vs. T1‐2)	2	892	1.01 (0.40,2.55)	0.979	87.0	0.006
TNM stage (III–IV vs. I–II)	5	2664	1.09 (0.70,1.72)	0.696	79.9	< 0.001
Lymph node metastasis (yes vs. no)	2	892	0.89 (0.39,2.01)	0.781	83.3	0.015
MSI (stable vs. unstable)	3	1433	0.56 (0.31,1.02)	0.057	69.7	0.037
Unclear compartment TANs						
Age (older vs. younger)	3	971	0.83 (0.56,1.22)	0.346	60.3	0.081
Gender (male vs. female)	4	1192	0.73 (0.54,0.97)	0.032	43.5	0.150
Tumor location (colon vs. rectum)	3	971	1.31 (1.00,1.72)	0.049	0	0.543
Primary tumor site (left colon vs. right colon)	2	253	0.56 (0.34,0.93)	0.026	0	0.661
Differential grade(G3 vs. G1‐2)	2	801	0.52 (0.15,1.79)	0.299	84.1	0.002
T stage (T3‐4 vs. T1‐2)	2	556	1.20 (0.77,1.85)	0.425	0	0.712
TNM stage (III–IV vs. I–II)	4	1192	0.99 (0.71,1.39)	0.959	60.2	0.057
Lymph node metastasis (N1‐2 vs. N0)	2	558	1.30 (0.98,1.71)	0.067	0	0.388
CEA (elevated vs. normal)	2	696	1.09 (0.83,1.43)	0.539	0	0.456
CA199 (elevated vs. normal)	2	696	1.08 (0.78,1.52)	0.630	0	0.601

Abbreviations: CI, confidence interval; OR, odds ratio; TNM, tumor‐node‐metastasis; CEA, carcinoembryonic antigen; CA199, carbohydrate antigen 199; MSI, microsatellite instability.

### 3.7 Publication Bias and Sensitivity Analysis

Funnel plots and Egger's tests were used to evaluate publication bias. No publication bias was observed (*p* = 0.063 for Egger's test) in five studies reporting the prognostic value of unclear compartment TANs on OS (Figure S1).

Our sensitivity analysis showed that all data assessing the association between IT, PT, and unclear compartment TANs and different outcomes (e.g., OS, DFS, and CSS) of CRC patients were stable (Figure S2).

## 4 Discussion

In this meta‐analysis, high TAN infiltration tended to be associated with better prognoses for CRC patients, although statistical significance was only observed between PT TANs and CSS. Differences in the subgroups of antibody type, tumor stage, quantitative methods, and follow‐up time were found for DFS. High TAN infiltration was correlated with mucinous histology type, female sex, colon cancer, and right‐sided colon cancer.

A previous meta‐analysis of the prognostic value of TANs in cancer suggested that high levels of IT neutrophils were associated with a worse prognosis [20], which was inconsistent with our results. However, this previous meta‐analysis included only two CRC studies [21, 22]. Increasing studies have investigated TANs in CRC. Our meta‐analysis updated the prognostic value of TANs in CRC. However, the association remains controversial.

Different reasons may explain the discrepancies in findings between these studies.

The differences in the phenotype and location of neutrophils in tumors may largely explain the different prognostic values of TANs in CRC. Two phenotypes of TANs have been identified: the N1 type exhibits an anti‐tumor function, and the N2 type plays a pro‐tumor role [43]. The phenotypic differentiation of TANs is regulated by cytokines in the TME [12]. Interestingly, we found a clear difference in the prognostic value of TANs among CRC patients when TANs were quantified by genetic analysis or directly in tumor tissues. High TAN expression at the gene level was associated with poor CRC prognosis [38, 44, 45, 46], while the prognostic value of TANs in CRC detected by immunohistochemistry or H&E staining varied, which may be explained by the above statement that TANs differentiate into different phenotypes when induced by different factors and therefore exert anti‐tumor or pro‐tumor effects. The location of TAN infiltration is also involved in the phenotypic differentiation of TANs and tumor progression. In the early‐stage tumors, TANs mainly infiltrate the tumor margin and exhibit an N1 phenotype, whereas in late‐stage tumors, TANs mainly infiltrate the tumor center and exhibit an N2 phenotype [10]. Many studies have found that IT TAN infiltration was more significantly associated with poor prognosis than PT TAN infiltration [34, 47, 48], which was inconsistent with our results that high TAN infiltration, whether in the IT or PT compartment, tended to be associated with better prognosis. No statistical difference was detected in our subgroup analysis of the TAN location. More precise evidence is needed to determine the phenotypic differentiation and role of neutrophils in tumor progression.

Another explanation could be the highly heterogeneous behavior of CRC. Inflammatory infiltration may differ among molecular subtypes of CRC [3], even in CRC at the same location or with the same tumor stage, leading to differences in the phenotypic differentiation of TANs. This phenomenon of inconsistent TAN prognostic directions in CRC was also found in gastric cancer [49, 50]. The gender of patients and tumor location may also contribute to the prognosis of CRC patients, as male CRC patients or patients with right‐sided colon cancer have poor prognoses [51]. Our results, that high TAN infiltration was associated with female patients and right‐sided colon cancer, seem conflicting.

The different methods used to measure and quantify TANs may also contribute to the diverse prognostic directions of TANs in CRC. Our meta‐analysis suggests a possible variation in the prognostic value of TANs with different measurement and quantitative methods. CD15 has been commonly used to label TANs, and now CD66b is widely used to label TANs. However, neither CD15 nor CD66b recognizes different TAN phenotypes, resulting in diverse prognostic values and conflicting prognostic directions. The methods used to quantify TANs also varied between studies. Automatic computerized quantification minimizes inter‐observer variability compared with stereological and semiquantitative methods. However, most of the studies included in this meta‐analysis used stereological and semiquantitative methods, and this may explain the differences in evaluating the prognostic value of TANs in CRC patients among the studies. It is crucial to unify the measurement and quantification methods to further distinguish the homogeneity and heterogeneity of TANs in CRC to address their differential prognostic value in CRC.

The importance of intestinal microbiota in the occurrence, progression, and metastasis of CRC has been widely reported [52, 53]. The intestinal microbiome differs by geographic location, ethnicity, and individual dietary habits and lifestyle. Many studies have confirmed differences in the intestinal microbiota between different ethnic groups, which may be due to genetic differences and inheritance [54, 55]. Differences in the genetic levels of intestinal microbiota between Chinese, American, and European races have also been reported [56]. The results of our subgroup analysis showing that the prognostic value of high TAN infiltration was more prominent in non‐Asian CRC patients partially confirmed this racial difference and might provide some clues for different CRC treatments in different ethnic groups in the future.

Finally, several studies have explored the relationship between neutrophil infiltration and chemotherapeutic efficiency in CRC [29, 36, 38, 42]. High TANs infiltration could improve the prognosis of CRC patients with adjuvant chemotherapy, but not those without adjuvant chemotherapy. High TAN infiltration could be an effective predictor of response to chemotherapy in CRC patients. More evidence is needed to confirm this possibility for further clinical application. However, the above results were contrary to the findings in blood. A lower pretreatment blood neutrophil–lymphocyte ratio (NLR) was associated with better tumor remission in CRC patients after chemotherapy or neoadjuvant radiotherapy [57, 58]. The NLR, a systemic inflammatory marker routinely obtained in cancer patients, has received great attention and has been widely studied as a new and effective prognostic marker for cancer. An elevated NLR was associated with poor prognosis and tumor recurrence in CRC patients [59, 60, 61, 62]. The prognostic differences between the blood NLR and TANs may be partly explained by the differentiation of neutrophils into different phenotypes in response to various factors. A possible association between a high NLR in the circulating blood and high TAN infiltration in tumors has been proposed [63], although sufficient studies are lacking. The combination of the NLR in circulating blood and TANs in the tumor may better predict the prognoses of CRC patients [63]. However, more studies are needed to confirm this possibility.

To our knowledge, this was the first study to conduct a separate meta‐analysis and systematic review of the prognostic value of TANs in CRC. We evaluated the impact of each factor on the prognostic value of TANs in CRC, and all survival outcomes as comprehensively as possible.

Several limitations of this meta‐analysis deserve our attention. First, the location, measurement methods, quantitative methods, and cutoff criteria of TANs in each included study differed. The antibody types or dilution ratios also differed, even when the same immune markers were used. All these factors might contribute to the observed heterogeneity. Therefore, a unified standard needs to be established. Secondly, although subgroup analyses and sensitivity analyses were performed, identifying the potential sources of heterogeneity was difficult, possibly due to limitations in the number of studies and sample size. Thirdly, many studies with unadjusted data were included for comparability, which may have affected the reliability of the results. Finally, the majority of the studies we included were retrospective studies, and more prospective studies are needed to confirm the prognostic value of TANs.

## 5 Conclusion

High TAN infiltration in the peritumoral compartment was associated with a better prognosis in CRC patients, especially in CSS. More relevant studies are needed to further verify the results of this study and explore the phenotypic differences in TANs in CRC to provide new ideas for CRC immunotherapy.

## Author Contributions

**Mengyuan Jiang:** conceptualization (lead), data curation (lead), formal analysis (lead), methodology (lead), resources (lead), software (lead), supervision (equal), validation (equal), visualization (equal), writing – original draft (lead), writing – review and editing (lead). **Rui Zhang:** conceptualization (equal), data curation (equal), formal analysis (equal), investigation (equal), methodology (equal), supervision (equal), validation (equal), visualization (equal), writing – original draft (equal). **Min Huang:** formal analysis (equal), investigation (equal), supervision (equal), validation (equal). **Jing Yang:** supervision (equal), validation (equal). **Qianqian Liu:** validation (equal), visualization (equal). **Ziru Zhao:** supervision (equal), validation (equal). **Ya Ma:** supervision (equal), validation (equal). **Hongfan Zhao:** supervision (equal), validation (equal). **Min Zhang:** conceptualization (supporting), funding acquisition (lead), project administration (lead), supervision (supporting).

## Ethics Statement

The authors have nothing to report.

## Consent

The authors have nothing to report.

## Conflicts of Interest

The authors declare no conflicts of interest.

## Supporting information


Data S1.

## Data Availability

Detailed extraction data are available from the corresponding author on reasonable request.
