# Supplementary material for: The Prognostic Value of Tumor‐Associated Neutrophils in Colorectal Cancer: A Systematic Review and Meta‐Analysis
Source: Cancer Med. 2025 Feb 27;14(5):e70614. doi: 10.1002/cam4.70614 (PMC11865885; doi:10.1002/cam4.70614)
Supplement: Supplementary file 1 — Data S1. [file CAM4-14-e70614-s001.docx]

***Supplemental Materials***

**The prognostic value of tumor-associated neutrophils in colorectal cancer: A systematic review and meta-analysis**

**Mengyuan Jiang, Rui Zhan, Min Huang, Jing Yang, Qianqian Liu, Ziru Zhao, Ya Ma, Hongfan Zhao, Min Zhang***

*** Correspondence:** Min Zhang, M.D., PhD. Department of Scientific Research Office, Gansu Provincial Hospital. Email: sallyzhangmin@126.com.

**Supplementary Figures and Tables**

1. **Supplementary Table 1.** Complete Search Strategies in PubMed (1), Embase (2) and the Cochrane Library (3).
2. **Supplementary Table 2.** Characteristics of studies included in this meta-analysis.
3. **Supplementary Table 3.** Risk of bias assessment (QUIPS).
4. **Supplementary Figure 1.** Funnel plot of prognostic value of unclear compartment TANs on OS.
5. **Supplementary Figure 2.** Results of sensitivity analysis.

**Supplementary Table 1.** Complete Search Strategies

Search strategies designed and executed by Mengyuan Jiang.

**1. PubMed search strategy:**

= 810 results on 9/1/2023

((((("Colorectal Neoplasms"[Mesh]) OR "Colonic Neoplasms"[Mesh]) OR "Rectal Neoplasms"[Mesh]) OR (‘Colorectal Neoplasm’[Title/Abstract] OR ‘Colorectal Neoplasms’[Title/Abstract] OR ‘Neoplasm, Colorectal’[Title/Abstract] OR ‘Neoplasms, Colorectal’[Title/Abstract] OR ‘Colorectal Tumors’[Title/Abstract] OR ‘Colorectal Tumor’[Title/Abstract] OR ‘Tumor, Colorectal’[Title/Abstract] OR ‘Tumors, Colorectal’[Title/Abstract] OR ‘Colorectal Cancer’[Title/Abstract] OR ‘Cancer, Colorectal’[Title/Abstract] OR ‘Cancers, Colorectal’[Title/Abstract] OR ‘Colorectal Cancers’[Title/Abstract] OR ‘Colorectal Carcinoma’[Title/Abstract] OR ‘Carcinoma, Colorectal’[Title/Abstract] OR ‘Carcinomas, Colorectal’[Title/Abstract] OR ‘Colorectal Carcinomas’[Title/Abstract] OR ‘Colonic Neoplasm’[Title/Abstract] OR ‘Neoplasm, Colonic’[Title/Abstract] OR ‘Neoplasms, Colonic’[Title/Abstract] OR ‘Colon Neoplasms’[Title/Abstract] OR ‘Colon Neoplasm’[Title/Abstract] OR ‘Neoplasm, Colon’[Title/Abstract] OR ‘Neoplasms, Colon’[Title/Abstract] OR ‘Cancer of Colon’[Title/Abstract] OR ‘Colon Cancers’[Title/Abstract] OR ‘Colon Cancer’[Title/Abstract] OR ‘Cancer, Colon’[Title/Abstract] OR ‘Cancers, Colon’[Title/Abstract] OR ‘Cancer of the Colon’[Title/Abstract] OR ‘Colonic Cancer’[Title/Abstract] OR ‘Cancer, Colonic’[Title/Abstract] OR ‘Cancers, Colonic’[Title/Abstract] OR ‘Colonic Cancers’[Title/Abstract] OR ‘Colon Adenocarcinoma’[Title/Abstract] OR ‘Adenocarcinoma, Colon’[Title/Abstract] OR ‘Adenocarcinomas, Colon’[Title/Abstract] OR ‘Colon Adenocarcinomas’[Title/Abstract] OR ‘Neoplasm, Rectal’[Title/Abstract] OR ‘Rectal Neoplasm’[Title/Abstract] OR ‘Rectum Neoplasms’[Title/Abstract] OR ‘Neoplasm, Rectum’[Title/Abstract] OR ‘Rectum Neoplasm’[Title/Abstract] OR ‘Rectal Tumors’[Title/Abstract] OR ‘Rectal Tumor’[Title/Abstract] OR ‘Tumor, Rectal’[Title/Abstract] OR ‘Neoplasms, Rectal’[Title/Abstract] OR ‘Cancer of Rectum’[Title/Abstract] OR ‘Rectum Cancers’[Title/Abstract] OR ‘Rectal Cancer’[Title/Abstract] OR ‘Cancer, Rectal’[Title/Abstract] OR ‘Rectal Cancers’[Title/Abstract] OR ‘Rectum Cancer’[Title/Abstract] OR ‘Cancer, Rectum’[Title/Abstract] OR ‘Cancer of the Rectum’[Title/Abstract] OR ‘colorectal neoplasia’[Title/Abstract] OR ‘colorectal tumorigenesis’[Title/Abstract] OR ‘colorectal tumour’[Title/Abstract] OR ‘neoplastic colorectal’[Title/Abstract] OR ‘tumour, colorectal’[Title/Abstract] OR ‘colon mass’[Title/Abstract] OR ‘colon neoplasia’[Title/Abstract] OR ‘colon tumorigenesis’[Title/Abstract] OR ‘colon tumour’[Title/Abstract] OR ‘colon villous tumor’[Title/Abstract] OR ‘colon villous tumour’[Title/Abstract] OR ‘colonic mass’[Title/Abstract] OR ‘colonic tumor’[Title/Abstract] OR ‘colonic masses’[Title/Abstract] OR ‘colonic tumors’[Title/Abstract] OR ‘colonic neoplasia’[Title/Abstract] OR ‘colonic tumorigenesis’[Title/Abstract] OR ‘colonic tumour’[Title/Abstract] OR ‘mesocolon tumor’[Title/Abstract] OR ‘mesocolon tumour’[Title/Abstract] OR ‘neoplasia in the colon’[Title/Abstract] OR ‘neoplasia of the colon’[Title/Abstract] OR ‘neoplastic colon’[Title/Abstract] OR ‘neoplastic colonic’[Title/Abstract] OR ‘tumor in the colon; ’[Title/Abstract] OR ‘tumor of the colon’[Title/Abstract] OR ‘tumorigenesis in colon’[Title/Abstract] OR ‘cancer of the colon’[Title/Abstract] OR ‘colon malignancies’[Title/Abstract] OR ‘colon malignancy’[Title/Abstract] OR ‘colonic cancer’[Title/Abstract] OR ‘colonic malignancies’[Title/Abstract] OR ‘colonic malignancy’[Title/Abstract] OR ‘malignancies of the colon’[Title/Abstract] OR ‘malignancy of the colon’[Title/Abstract] OR ‘malignant colon tumor’[Title/Abstract] OR ‘malignant neoplasm of the colon’[Title/Abstract] OR ‘malignant tumor of the colon’[Title/Abstract] OR ‘carcinoma of colon and rectum’[Title/Abstract] OR ‘carcinoma of rectum and colon’[Title/Abstract] OR ‘carcinoma of the colon and the rectum’[Title/Abstract] OR ‘carcinoma of the rectum and the colon’[Title/Abstract] OR ‘carcinomatous colorectal’[Title/Abstract] OR ‘colo-rectal carcinoma’[Title/Abstract] OR ‘colorectal carcinomata’[Title/Abstract] OR ‘colorectal carcinomatosis’[Title/Abstract] OR ‘rectocolonic carcinoma’[Title/Abstract] OR ‘carcinoma coli’[Title/Abstract] OR ‘carcinoma in the colon’[Title/Abstract] OR ‘carcinoma of the colon’[Title/Abstract] OR ‘carcinomata of the colon’[Title/Abstract] OR ‘carcinomatous colon’[Title/Abstract] OR ‘colon carcinoma diagnosis’[Title/Abstract] OR ‘colon carcinomatosis’[Title/Abstract] OR ‘colonic carcinoma’[Title/Abstract] OR ‘colonic carcinomata’[Title/Abstract] OR ‘carcinoma of the lower rectum’[Title/Abstract] OR ‘carcinoma of the middle rectum’[Title/Abstract] OR ‘carcinoma of the upper rectum’[Title/Abstract] OR ‘carcinoma recti’[Title/Abstract] OR ‘chronic carcinoma recti’[Title/Abstract] OR ‘chronic rectal carcinoma’[Title/Abstract] OR ‘chronic rectum carcinoma’[Title/Abstract] OR ‘hard carcinoma recti’[Title/Abstract] OR ‘hard rectal carcinoma’[Title/Abstract] OR ‘hard rectum carcinoma’[Title/Abstract] OR ‘rectal carcinoma’[Title/Abstract] OR ‘rectal chronic carcinoma’[Title/Abstract] OR ‘rectal hard carcinoma’[Title/Abstract] OR ‘rectum ampulla carcinoma’[Title/Abstract] OR ‘rectum chronic carcinoma’[Title/Abstract] OR ‘rectum hard carcinoma’[Title/Abstract] OR ‘mass, rectum (tumor) ’[Title/Abstract] OR ‘neoplasm of the rectum’[Title/Abstract] OR ‘neoplasma recti’[Title/Abstract] OR ‘pararectal tumor’[Title/Abstract] OR ‘pararectal tumour’[Title/Abstract] OR ‘rectal mass’[Title/Abstract] OR ‘rectal neoplasia’[Title/Abstract] OR ‘rectal tumour’[Title/Abstract] OR ‘rectum mass ’[Title/Abstract] OR ‘rectum tumor’[Title/Abstract] OR ‘rectum neoplasia’[Title/Abstract] OR ‘rectum tumour’[Title/Abstract] OR ‘retrorectal tumor’[Title/Abstract] OR ‘retrorectal tumour’[Title/Abstract] OR ‘tumor of the rectum’[Title/Abstract] OR ‘tumor recti’[Title/Abstract] OR ‘tumour of the rectum’[Title/Abstract] OR ‘tumour recti’[Title/Abstract] OR ‘cancer of the lower rectum’[Title/Abstract] OR ‘cancer of the rectum’[Title/Abstract] OR ‘cancer of the upper rectum’[Title/Abstract] OR ‘malignancies of the rectum’[Title/Abstract] OR ‘malignancy, rectum’[Title/Abstract] OR ‘rectal carcinogenesis’[Title/Abstract] OR ‘rectal malignancies’[Title/Abstract] OR ‘rectal malignancy’[Title/Abstract] OR ‘rectum malignancy’[Title/Abstract] OR ‘cancer of colon[Title/Abstract] OR ‘cancer of rectum’[Title/Abstract] OR ‘cancer of the colon and the rectum’[Title/Abstract] OR ‘cancer of the rectum and the colon’[Title/Abstract] OR ‘colo-rectal cancer’[Title/Abstract] OR ‘colo-rectal carcinogenesis’[Title/Abstract] OR ‘colo-rectal malignancies’[Title/Abstract] OR ‘colo-rectal malignancy’[Title/Abstract] OR ‘colorectal cancerogenesis’[Title/Abstract] OR ‘colorectal carcinogenesis’[Title/Abstract] OR ‘colorectal malignancies’[Title/Abstract] OR ‘colorectal malignancy’[Title/Abstract] OR ‘malignancies of the colon and rectum’[Title/Abstract] OR ‘malignancies of colon and rectum’[Title/Abstract] OR ‘malignancy of colon and rectum’[Title/Abstract] OR ‘malignancy of the colon and rectum’[Title/Abstract] OR ‘recto-colonic cancer’[Title/Abstract] OR ‘rectocolonic cancer’[Title/Abstract])) AND (("Neutrophils"[Mesh]) OR (Neutrophils[Title/Abstract] OR Neutrophil[Title/Abstract] OR ‘Polymorphonuclear Leukocyte’[Title/Abstract] OR ‘Polymorphonuclear Leukocytes’[Title/Abstract] OR ‘Polymorphonuclear Neutrophils’[Title/Abstract] OR ‘Polymorphonuclear Neutrophil’[Title/Abstract] OR ‘polymorphonuclear granulocyte’[Title/Abstract] OR ‘polymorphonuclear leucocyte’[Title/Abstract] OR ‘polymorphous leucocyte’[Title/Abstract] OR ‘polymorphous leukocyte’[Title/Abstract] OR ‘polynuclear leucocyte’[Title/Abstract] OR ‘polynuclear leukocyte’[Title/Abstract] OR ‘LE Cells’[Title/Abstract] OR ‘LE Cell’[Title/Abstract] OR ‘Neutrophil Band Cells’[Title/Abstract] OR ‘Neutrophil Band Cell’[Title/Abstract] OR ‘tumor-infiltrating neutrophils’[Title/Abstract] OR ‘tumor-associated neutrophils’[Title/Abstract] OR neutrocyte[Title/Abstract] OR neutrocytes[Title/Abstract] OR ‘neutrophil granulocyte’[Title/Abstract] OR ‘neutrophil leucocyte’[Title/Abstract] OR ‘neutrophil leukocyte’[Title/Abstract] OR ‘neutrophilic granulocyte’[Title/Abstract] OR ‘neutrophilic leucocyte’[Title/Abstract] OR ‘neutrophilic leukocyte’[Title/Abstract] OR ‘pmn granulocyte’[Title/Abstract] OR ‘pmn leucocyte’[Title/Abstract] OR ‘pmn leukocyte’[Title/Abstract] OR ‘pmn neutrophil’[Title/Abstract]))) AND (("Prognosis"[Mesh]) OR (Prognosis[Title/Abstract] OR Prognoses[Title/Abstract] OR ‘Prognostic Factors’[Title/Abstract] OR ‘Prognostic Factor’[Title/Abstract] OR ‘clinical outcome’[Title/Abstract]))

**Total 810**

**2. Embase search strategy:**

= 1368 results on 9/1/2023

('colorectal cancer'/exp OR 'colorectal tumor'/exp OR 'colorectal carcinoma'/exp OR 'rectum cancer'/exp OR 'rectum tumor'/exp OR 'rectum carcinoma'/exp OR 'colon cancer'/exp OR 'colon tumor'/exp OR 'colon carcinoma'/exp OR 'colorectal neoplasm':ab,ti OR 'neoplasm, colorectal':ab,ti OR 'neoplasms, colorectal':ab,ti OR 'colorectal tumors':ab,ti OR 'colorectal tumor':ab,ti OR 'tumor, colorectal':ab,ti OR 'tumors, colorectal':ab,ti OR 'colorectal cancer':ab,ti OR 'cancer, colorectal':ab,ti OR 'cancers, colorectal':ab,ti OR 'colorectal cancers':ab,ti OR 'colorectal carcinoma':ab,ti OR 'carcinoma, colorectal':ab,ti OR 'carcinomas, colorectal':ab,ti OR 'colorectal carcinomas':ab,ti OR 'colonic neoplasm':ab,ti OR 'neoplasm, colonic':ab,ti OR 'neoplasms, colonic':ab,ti OR 'colon neoplasms':ab,ti OR 'colon neoplasm':ab,ti OR 'neoplasm, colon':ab,ti OR 'neoplasms, colon':ab,ti OR 'cancer of colon':ab,ti OR 'colon cancers':ab,ti OR 'colon cancer':ab,ti OR 'cancer, colon':ab,ti OR 'cancers, colon':ab,ti OR 'cancer, colonic':ab,ti OR 'cancers, colonic':ab,ti OR 'colonic cancers':ab,ti OR 'colon adenocarcinoma':ab,ti OR 'adenocarcinoma, colon':ab,ti OR 'adenocarcinomas, colon':ab,ti OR 'colon adenocarcinomas':ab,ti OR 'neoplasm, rectal':ab,ti OR 'rectal neoplasm':ab,ti OR 'rectum neoplasms':ab,ti OR 'neoplasm, rectum':ab,ti OR 'rectum neoplasm':ab,ti OR 'rectal tumors':ab,ti OR 'rectal tumor':ab,ti OR 'tumor, rectal':ab,ti OR 'neoplasms, rectal':ab,ti OR 'cancer of rectum':ab,ti OR 'rectum cancers':ab,ti OR 'rectal cancer':ab,ti OR 'cancer, rectal':ab,ti OR 'rectal cancers':ab,ti OR 'rectum cancer':ab,ti OR 'cancer, rectum':ab,ti OR 'colorectal neoplasia':ab,ti OR 'colorectal tumorigenesis':ab,ti OR 'colorectal tumour':ab,ti OR 'neoplastic colorectal':ab,ti OR 'tumour, colorectal':ab,ti OR 'colon mass':ab,ti OR 'colon neoplasia':ab,ti OR 'colon tumorigenesis':ab,ti OR 'colon tumour':ab,ti OR 'colon villous tumor':ab,ti OR 'colon villous tumour':ab,ti OR 'colonic mass':ab,ti OR 'colonic masses':ab,ti OR 'colonic tumors':ab,ti OR 'colonic neoplasia':ab,ti OR 'colonic tumor':ab,ti OR 'colonic tumorigenesis':ab,ti OR 'colonic tumour':ab,ti OR 'mesocolon tumor':ab,ti OR 'mesocolon tumour':ab,ti OR 'neoplasia in the colon':ab,ti OR 'neoplasia of the colon':ab,ti OR 'neoplastic colon':ab,ti OR 'neoplastic colonic':ab,ti OR 'tumor in the colon;':ab,ti OR 'tumor of the colon':ab,ti OR 'tumorigenesis in colon':ab,ti OR 'cancer of the colon':ab,ti OR 'colon malignancies':ab,ti OR 'colon malignancy':ab,ti OR 'colonic cancer':ab,ti OR 'colonic malignancies':ab,ti OR 'colonic malignancy':ab,ti OR 'malignancies of the colon':ab,ti OR 'malignancy of the colon':ab,ti OR 'malignant colon tumor':ab,ti OR 'malignant neoplasm of the colon':ab,ti OR 'malignant tumor of the colon':ab,ti OR 'carcinoma of colon and rectum':ab,ti OR 'carcinoma of rectum and colon':ab,ti OR 'carcinoma of the colon and rectum':ab,ti OR 'carcinoma of the colon and the rectum':ab,ti OR 'carcinoma of the rectum and colon':ab,ti OR 'carcinoma of the rectum and the colon':ab,ti OR 'carcinomatous colorectal':ab,ti OR 'colo-rectal carcinoma':ab,ti OR 'colorectal carcinomata':ab,ti OR 'colorectal carcinomatosis':ab,ti OR 'rectocolonic carcinoma':ab,ti OR 'carcinoma coli':ab,ti OR 'carcinoma in the colon':ab,ti OR 'carcinoma of the colon':ab,ti OR 'carcinomata of the colon':ab,ti OR 'carcinomatous colon':ab,ti OR 'colon carcinoma diagnosis':ab,ti OR 'colon carcinomatosis':ab,ti OR 'colonic carcinoma':ab,ti OR 'colonic carcinomata':ab,ti OR 'carcinoma of the lower rectum':ab,ti OR 'carcinoma of the middle rectum':ab,ti OR 'carcinoma of the upper rectum':ab,ti OR 'carcinoma recti':ab,ti OR 'chronic carcinoma recti':ab,ti OR 'chronic rectal carcinoma':ab,ti OR 'chronic rectum carcinoma':ab,ti OR 'hard carcinoma recti':ab,ti OR 'hard rectal carcinoma':ab,ti OR 'hard rectum carcinoma':ab,ti OR 'rectal carcinoma':ab,ti OR 'rectal chronic carcinoma':ab,ti OR 'rectal hard carcinoma':ab,ti OR 'rectum ampulla carcinoma':ab,ti OR 'rectum chronic carcinoma':ab,ti OR 'rectum hard carcinoma':ab,ti OR 'mass, rectum tumor':ab,ti OR 'neoplasm of the rectum':ab,ti OR 'neoplasma recti':ab,ti OR 'pararectal tumor':ab,ti OR 'pararectal tumour':ab,ti OR 'rectal mass':ab,ti OR 'rectal neoplasia':ab,ti OR 'rectal tumour':ab,ti OR 'rectum mass':ab,ti OR 'rectum tumor':ab,ti OR 'rectum neoplasia':ab,ti OR 'rectum tumour':ab,ti OR 'retrorectal tumor':ab,ti OR 'retrorectal tumour':ab,ti OR 'tumor of the rectum':ab,ti OR 'tumor recti':ab,ti OR 'tumour of the rectum':ab,ti OR 'tumour recti':ab,ti OR 'cancer of the lower rectum':ab,ti OR 'cancer of the rectum':ab,ti OR 'cancer of the upper rectum':ab,ti OR 'malignancies of the rectum':ab,ti OR 'malignancy, rectum':ab,ti OR 'rectal carcinogenesis':ab,ti OR 'rectal malignancies':ab,ti OR 'rectal malignancy':ab,ti OR 'rectum malignancy':ab,ti OR 'cancer of colon and rectum':ab,ti OR 'cancer of rectum and colon':ab,ti OR 'cancer of the colon and rectum':ab,ti OR 'cancer of the colon and the rectum':ab,ti OR 'cancer of the rectum and colon':ab,ti OR 'cancer of the rectum and the colon':ab,ti OR 'colo-rectal cancer':ab,ti OR 'colo-rectal carcinogenesis':ab,ti OR 'colo-rectal malignancies':ab,ti OR 'colo-rectal malignancy':ab,ti OR 'colorectal cancerogenesis':ab,ti OR 'colorectal carcinogenesis':ab,ti OR 'colorectal malignancies':ab,ti OR 'colorectal malignancy':ab,ti OR 'malignancies of the colon and rectum':ab,ti OR 'malignancy of colon and rectum':ab,ti OR 'malignancy of the colon and rectum':ab,ti OR 'recto-colonic cancer':ab,ti OR 'rectocolonic cancer':ab,ti) AND ('neurophil'/exp OR neutrophils:ab,ti OR neutrophil:ab,ti OR 'polymorphonuclear leukocyte':ab,ti OR 'polymorphonuclear leukocytes':ab,ti OR 'polymorphonuclear neutrophils':ab,ti OR 'polymorphonuclear neutrophil':ab,ti OR 'polymorphonuclear granulocyte':ab,ti OR 'polymorphonuclear leucocyte':ab,ti OR 'polymorphous leucocyte':ab,ti OR 'polymorphous leukocyte':ab,ti OR 'polynuclear leucocyte':ab,ti OR 'polynuclear leukocyte':ab,ti OR 'le cells':ab,ti OR 'le cell':ab,ti OR 'neutrophil band cells':ab,ti OR 'neutrophil band cell':ab,ti OR 'tumor-infiltrating neutrophils':ab,ti OR 'tumor-associated neutrophils':ab,ti OR neutrocyte:ab,ti OR neutrocytes:ab,ti OR 'neutrophil granulocyte':ab,ti OR 'neutrophil leucocyte':ab,ti OR 'neutrophil leukocyte':ab,ti OR 'neutrophilic granulocyte':ab,ti OR 'neutrophilic leucocyte':ab,ti OR 'neutrophilic leukocyte':ab,ti OR 'pmn granulocyte':ab,ti OR 'pmn leucocyte':ab,ti OR 'pmn leukocyte':ab,ti OR 'pmn neutrophil':ab,ti) AND ('prognosis'/exp OR prognosis:ab,ti OR prognoses:ab,ti OR 'prognostic factors':ab,ti OR 'prognostic factor':ab,ti OR 'clinical outcome':ab,ti)

**Total 1368**

**3. The Cochrane Library search strategy:**

= 235 results on 9/1/2023

ID Search

#1 MeSH descriptor: [Neutrophils] explode all trees

#2 MeSH descriptor: [Prognosis] explode all trees

#3 MeSH descriptor: [Colorectal Neoplasms] explode all trees

#4 MeSH descriptor: [Colonic Neoplasms] explode all trees

#5 MeSH descriptor: [Rectal Neoplasms] explode all trees

#6 (Prognosis OR Prognoses OR ‘Prognostic Factors’ OR ‘Prognostic Factor’ OR ‘clinical outcome’):ti,ab,kw

#7 (Neutrophils OR Neutrophil OR ‘Polymorphonuclear Leukocyte’ OR ‘Polymorphonuclear Leukocytes’ OR ‘Polymorphonuclear Neutrophils’ OR ‘Polymorphonuclear Neutrophil’ OR ‘polymorphonuclear granulocyte’ OR ‘polymorphonuclear leucocyte’ OR ‘polymorphous leucocyte’ OR ‘polymorphous leukocyte’ OR ‘polynuclear leucocyte’ OR ‘polynuclear leukocyte’ OR ‘LE Cells’ OR ‘LE Cell’ OR ‘Neutrophil Band Cells’ OR ‘Neutrophil Band Cell’ OR ‘tumor-infiltrating neutrophils’ OR ‘tumor-associated neutrophils’ OR neutrocyte OR neutrocytes OR ‘neutrophil granulocyte’ OR ‘neutrophil leucocyte’ OR ‘neutrophil leukocyte’ OR ‘neutrophilic granulocyte’ OR ‘neutrophilic leucocyte’ OR ‘neutrophilic leukocyte’ OR ‘pmn granulocyte’ OR ‘pmn leucocyte’ OR ‘pmn leukocyte’ OR ‘pmn neutrophil’):ti,ab,kw

#8 (‘Colorectal Neoplasm’ OR ‘Colorectal Neoplasms’ OR ‘Neoplasm, Colorectal’ OR ‘Neoplasms, Colorectal’ OR ‘Colorectal Tumors’ OR ‘Colorectal Tumor’ OR ‘Tumor, Colorectal’ OR ‘Tumors, Colorectal’ OR ‘Colorectal Cancer’ OR ‘Cancer, Colorectal’ OR ‘Cancers, Colorectal’ OR ‘Colorectal Cancers’ OR ‘Colorectal Carcinoma’ OR ‘Carcinoma, Colorectal’ OR ‘Carcinomas, Colorectal’ OR ‘Colorectal Carcinomas’ OR ‘Colonic Neoplasm’ OR ‘Neoplasm, Colonic’ OR ‘Neoplasms, Colonic’ OR ‘Colon Neoplasms’ OR ‘Colon Neoplasm’ OR ‘Neoplasm, Colon’ OR ‘Neoplasms, Colon’ OR ‘Cancer of Colon’ OR ‘Colon Cancers’ OR ‘Colon Cancer’ OR ‘Cancer, Colon’ OR ‘Cancers, Colon’ OR ‘Cancer of the Colon’ OR ‘Colonic Cancer’ OR ‘Cancer, Colonic’ OR ‘Cancers, Colonic’ OR ‘Colonic Cancers’ OR ‘Colon Adenocarcinoma’ OR ‘Adenocarcinoma, Colon’ OR ‘Adenocarcinomas, Colon’ OR ‘Colon Adenocarcinomas’ OR ‘Neoplasm, Rectal’ OR ‘Rectal Neoplasm’ OR ‘Rectum Neoplasms’ OR ‘Neoplasm, Rectum’ OR ‘Rectum Neoplasm’ OR ‘Rectal Tumors’ OR ‘Rectal Tumor’ OR ‘Tumor, Rectal’ OR ‘Neoplasms, Rectal’ OR ‘Cancer of Rectum’ OR ‘Rectum Cancers’ OR ‘Rectal Cancer’ OR ‘Cancer, Rectal’ OR ‘Rectal Cancers’ OR ‘Rectum Cancer’ OR ‘Cancer, Rectum’ OR ‘Cancer of the Rectum’ OR ‘colorectal neoplasia’ OR ‘colorectal tumorigenesis’ OR ‘colorectal tumour’ OR ‘neoplastic colorectal’ OR ‘tumour, colorectal’ OR ‘colon mass’ OR ‘colon neoplasia’ OR ‘colon tumorigenesis’ OR ‘colon tumour’ OR ‘colon villous tumor’ OR ‘colon villous tumour’ OR ‘colonic mass’ OR ‘colonic tumor’ OR ‘colonic masses’ OR ‘colonic tumors’ OR ‘colonic neoplasia’ OR ‘colonic tumor’ OR ‘colonic tumorigenesis’ OR ‘colonic tumour’ OR ‘mesocolon tumor’ OR ‘mesocolon tumour’ OR ‘neoplasia in the colon’ OR ‘neoplasia of the colon’ OR ‘neoplastic colon’ OR ‘neoplastic colonic’ OR ‘tumor in the colon; ’ OR ‘tumor of the colon’ OR ‘tumorigenesis in colon’ OR ‘cancer of the colon’ OR ‘colon malignancies’ OR ‘colon malignancy’ OR ‘colonic cancer’ OR ‘colonic malignancies’ OR ‘colonic malignancy’ OR ‘malignancies of the colon’ OR ‘malignancy of the colon’ OR ‘malignant colon tumor’ OR ‘malignant neoplasm of the colon’ OR ‘malignant tumor of the colon’ OR ‘carcinoma of colon and rectum’ OR ‘carcinoma of rectum and colon’ OR ‘carcinoma of the colon and rectum’ OR ‘carcinoma of the colon and the rectum’ OR ‘carcinoma of the rectum and colon’ OR ‘carcinoma of the rectum and the colon’ OR ‘carcinomatous colorectal’ OR ‘colo-rectal carcinoma’ OR ‘colorectal carcinomata’ OR ‘colorectal carcinomatosis’ OR ‘rectocolonic carcinoma’ OR ‘carcinoma coli’ OR ‘carcinoma in the colon’ OR ‘carcinoma of the colon’ OR ‘carcinomata of the colon’ OR ‘carcinomatous colon’ OR ‘colon carcinoma diagnosis’ OR ‘colon carcinomatosis’ OR ‘colonic carcinoma’ OR ‘colonic carcinomata’ OR ‘carcinoma of the lower rectum’ OR ‘carcinoma of the middle rectum’ OR ‘carcinoma of the upper rectum’ OR ‘carcinoma recti’ OR ‘chronic carcinoma recti’ OR ‘chronic rectal carcinoma’ OR ‘chronic rectum carcinoma’ OR ‘hard carcinoma recti’ OR ‘hard rectal carcinoma’ OR ‘hard rectum carcinoma’ OR ‘rectal carcinoma’ OR ‘rectal chronic carcinoma’ OR ‘rectal hard carcinoma’ OR ‘rectum ampulla carcinoma’ OR ‘rectum chronic carcinoma’ OR ‘rectum hard carcinoma’ OR ‘mass, rectum (tumor) ’ OR ‘neoplasm of the rectum’ OR ‘neoplasma recti’ OR ‘pararectal tumor’ OR ‘pararectal tumour’ OR ‘rectal mass’ OR ‘rectal neoplasia’ OR ‘rectal tumour’ OR ‘rectum mass ’ OR ‘rectum tumor’ OR ‘rectum neoplasia’ OR ‘rectum tumour’ OR ‘retrorectal tumor’ OR ‘retrorectal tumour’ OR ‘tumor of the rectum’ OR ‘tumor recti’ OR ‘tumour of the rectum’ OR ‘tumour recti’ OR ‘cancer of the lower rectum’ OR ‘cancer of the rectum’ OR ‘cancer of the upper rectum’ OR ‘malignancies of the rectum’ OR ‘malignancy, rectum’ OR ‘rectal carcinogenesis’ OR ‘rectal malignancies’ OR ‘rectal malignancy’ OR ‘rectum malignancy’ OR ‘cancer of colon and rectum’ OR ‘cancer of rectum and colon’ OR ‘cancer of the colon and rectum’ OR ‘cancer of the colon and the rectum’ OR ‘cancer of the rectum and colon’ OR ‘cancer of the rectum and the colon’ OR ‘colorectal cancer’ OR ‘colorectal carcinogenesis’ OR ‘colorectal malignancies’ OR ‘colorectal malignancy’ OR ‘colorectal cancerogenesis’ OR ‘colorectal carcinogenesis’ OR ‘colorectal malignancies’ OR ‘colorectal malignancy’ OR ‘malignancies of the colon and rectum’ OR ‘malignancy of colon and rectum’ OR ‘malignancy of the colon and rectum’ OR ‘recto-colonic cancer’ OR ‘rectocolonic cancer’):ti,ab,kw

#9 #1 OR #7

#10 #2 OR #6

#11 #3 OR #4 OR #5 OR #8

#12 #9 AND #10 AND #11

#9 AND #10 AND #11

**Total 235**

**Supplementary Table 2.** Characteristics of studies included in this meta-analysis

| **Study** | **Setting of population** | **Follow-up time (months) mean/ median (range)** | **Cutoff Value** | **Effect value** | **adjustments** |
| --- | --- | --- | --- | --- | --- |
| Xu, X. 2021 [36] | Hospital | Median 58 (interquartile range, 21–117) | 60 cells/0.1836 mm^2^ (50× magnification) | HR | FOXP3, CD163, tumor grade, TNM stage, Serum CEA, Serum CA199 |
| Berry, R. S. 2017 [24] | Hospital | Median 68 (0–250) | 5.4 cells/HPF | HR | None |
| Galdiero, M. R.^a^ 2016 [28] | Hospital | DSS：Mean 57.36 (SD= 33.36); DFS： Mean 52.56 (SD=35.04) | IM 0.1717%;  IT 0.11% | HR | None |
| Galdiero, M. R.^b^ 2016 [28] | Hospital | Mean 52.56 (SD=35.04) | IT 1.26% | HR | None |
| Ye, L.^a^ 2019 [37] | Hospital | NR | NR | HR | TNM stage, Differentiation grade, Number of lymph nodes |
| Ye, L.^b^ 2019 [37] | Hospital | NR | NR | HR | TNM stage, Chemotherapy, Number of metastatic lymph nodes, Serum CEA, Serum CA199 |
| Ye, L.^c^ 2019 [37] | Hospital | NR | NR | HR | Gender, Disease location, Differentiation grade, Number of metastatic lymph nodes |
| Zhu, B.^a^ 2018 [39] | Hospital | NR | 55 cells/field at 200× magnification | HR | CEA, Depth of invasion, Lymph node metastasis, Metastasis, IL-37, MMR status |
| Zhu, B.^b^ 2018 [39] | Hospital | NR | 55 cells/field at 200× magnification | HR | None |
| Rao, H. L. 2012 [19] | Hospital | Mean 55.42 (median 60.0; range, 0.5–98). | 60 cells per TMA spot | HR | pT status, pN status, pM status, Clinical stage |
| Rottmann, B. G. 2021 [33] | Hospital | Mean 45.6 (median 48; range, 0–108) | 15 cells per 100 tumor cells at 400× magnification (0.08 mm^2^) | HR | Age, Histological grade, TNM stage, MMR status |
| Klintrup, K. 2005 [30] | Hospital | Mean 41 months | Low: score (0-1); High: score (2-3) | HR | None |
| Governa, V. 2017 [29] | Hospital | NR | 10 cells per punch | HR | None |
| Richards, C. H. [20] | Hospital | Median 105 months (range, 55–163) | 15 cells per 0.018 mm^2^ | HR | None |
| Chen, Y. 2016 [25] | Hospital | Mean 62.9 (SD=29.3) | NR | HR | None |
| Zhu, Y. 2016 [40] | Hospital | DFS: Median 53 (range, 2–127); OS: Median 63 (range, 4–127) | IT： 154.9 cells/low power fields (×200);  IM： 177.4 cells/low power fields (×200) | HR | None |
| Mehrabi, S. F. 2021 [32] | Hospital | NR | NR | HR | Age, Gender, Lymph node metastasis, TNM stage, Tumor size, BDNF, CysLT1 |
| Lin, Y. 2015 [31] | Hospital | NR | 31 cells/HPF | RR | IL-17, Duke’s staging, Lymph node metastasis, Distant metastasis and recurrence |
| Wikberg, M. L. 2017 [34] | Hospital | NR | Low: score 1; High: score (2-4) | HR | Age, Sex, Grade, Stage, Tumor site |
| Xiong, Y. 2018 [35] | Database | NR | NR | HR | Age, Sex, Local invasion, Lymph node metastasis, Distant metastasis, TNM stage, Eosinophils, Dendritic cells activated, Macrophages M1, Macrophages M2 |
| Edin, S. 2019 [27] | Hospital | NR | l31.7 cells/mm^2^ (tumor compartments)  95.8 cells/mm^2^ (stromal compartments) | HR | One categorical immune parameter, Age, Localization, Stage, Sex |
| Zhou, G. 2018 [38] | Hospital | Median 66 (range, 1–82 months) | 8 cells/HPF | HR | TNM Stage, Differentiation grade, Age, Years, Gender, Site |
| Chengzeng, Y. 2022 [26] | Hospital | NR | 77 cells per HPF | HR | None |

CA: carbohydrate antigen; CEA: carcinoembryonic antigen; DFS: Disease-free survival; DSS: Disease-specific survival; FOXP3: forkhead box transcription factor; HPF: high power field; HR: hazard ratio; IM: invasion margin; IL: interleukin; IT: intratumoral; MMR: mismatch repair; PT: peritumoral; NR: not report; OS: Overall survival; RFS: Recurrence-free survival; ROC: receiver operating characteristic curve; SD: standard deviation; TANs: Tumor-associated neutrophils;TNM: tumor–node–metastasis; X-tile: X-tile bioinformatics software.

**Supplementary Table 3.** Risk of bias assessment (QUIPS)

| **Study** | **Study Participation** | **Study Attrition** | **Prognostic Factor Measurement** | **Outcome Measurement** | **Study Confounding** | **Statistical Analysis and Reporting** | **Overall** |
| --- | --- | --- | --- | --- | --- | --- | --- |
| Xu, X. 2021 | Low | Low | Low | Low | Low | Low | Low |
| Berry, R. S. 2017 | Low | Moderate | Low | Low | Moderate | Low | High |
| Galdiero, M. R. 2016 | Low | Low | Low | Low | Moderate | Low | Moderate |
| Ye, L. 2019 | Low | Low | Low | Low | Low | Low | Low |
| Zhu, B. 2018 | Low | Low | Low | Low | Low | Low | Low |
| Rao, H. L. 2012 | Low | Low | Low | Low | Low | Low | Low |
| Rottmann, B. G. 2021 | Low | Low | Low | Low | Low | Low | Low |
| Klintrup, K. 2005 | Low | Low | Moderate | Low | Moderate | Low | High |
| Governa, V. 2017 | Moderate | Low | Low | Low | Moderate | Low | High |
| Richards, C. H. 2012 | Low | Low | Low | Low | Moderate | Low | Moderate |
| Chen, Y. 2016 | Low | Low | Low | Low | Moderate | Low | Moderate |
| Zhu, Y. 2016 | Low | Low | Low | Low | Moderate | Low | Moderate |
| Mehrabi, S. F. 2021 | Moderate | Moderate | Low | Low | Low | Low | Low |
| Lin, Y. 2015 | Low | Low | Low | Low | Low | Low | Low |
| Wikberg, M. L. 2017 | Low | Moderate | Low | Low | Low | Low | Low |
| Xiong, Y. 2018 | Low | Low | Low | Low | Low | Low | Low |
| Edin, S. 2019 | Low | Low | Low | Moderate | Low | Low | Low |
| Zhou, G. 2018 | Low | Low | Low | Low | Low | Low | Low |
| Chengzeng, Y. 2022 | Low | Moderate | Low | Low | Moderate | Low | Moderate |


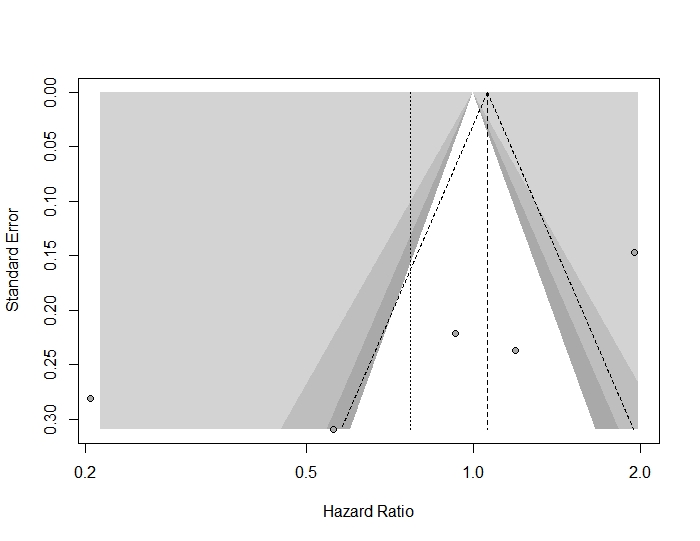


**Supplementary Figure 1.** Funnel plot of prognostic value of unclear compartment TANs on overall survival.

**A**

**
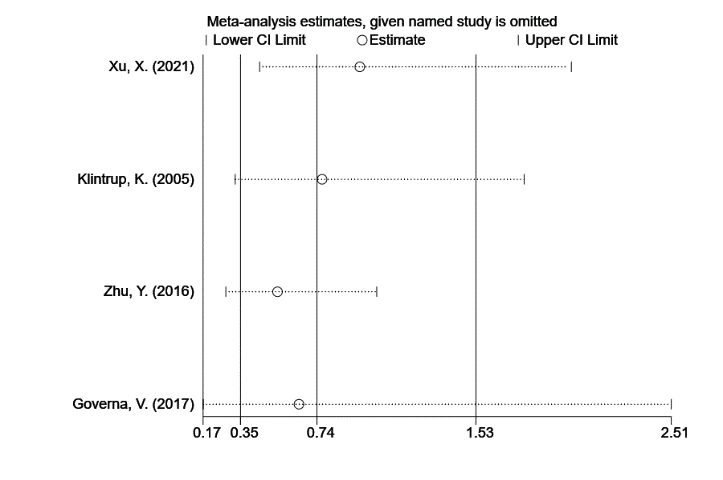
**

**B**

**
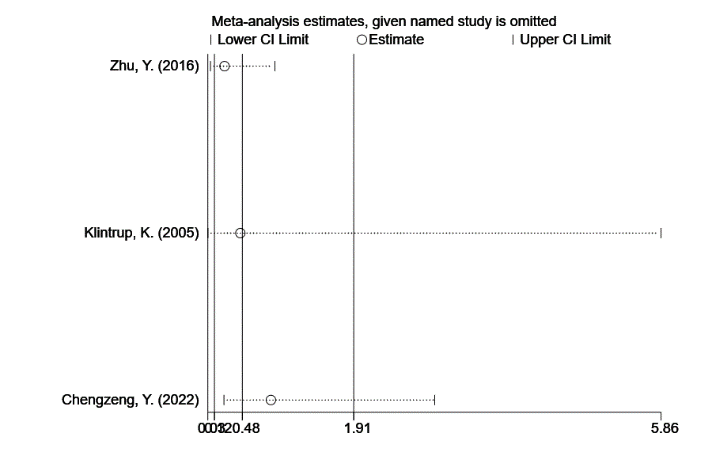
**

**C**

**
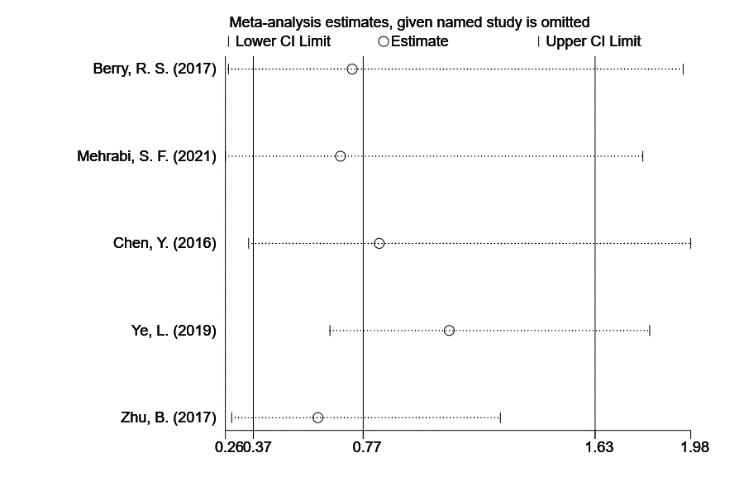
**

**D**

**
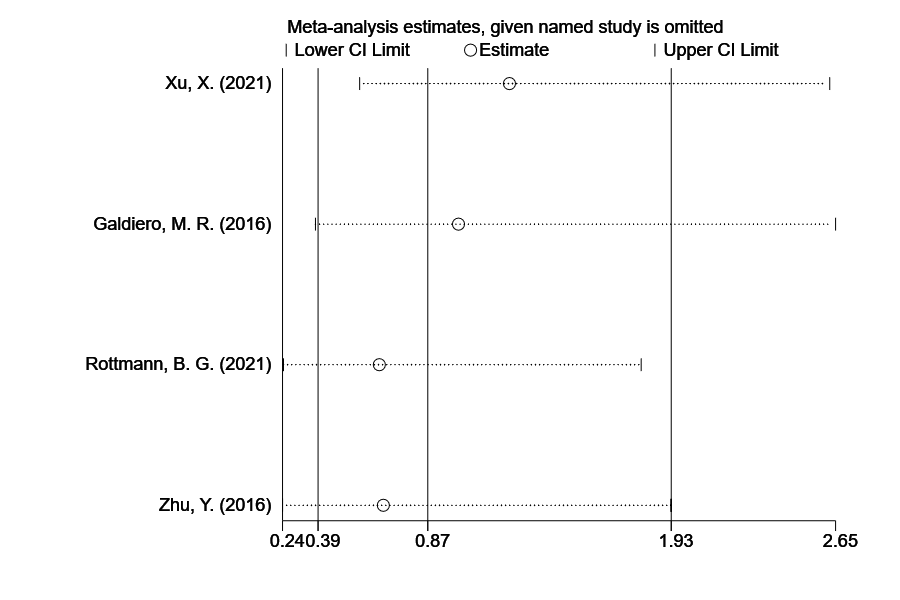
**

**E**

**
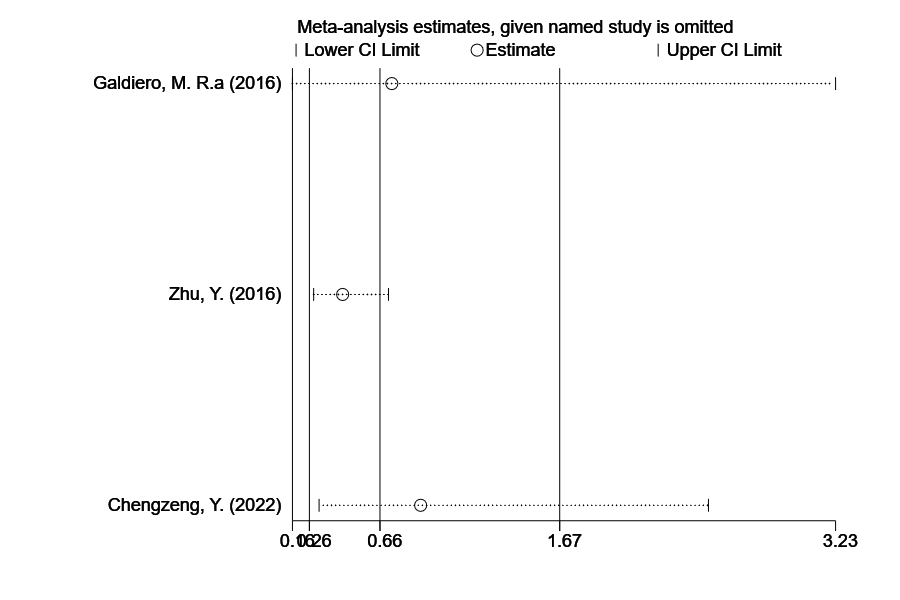
**

**F**

**
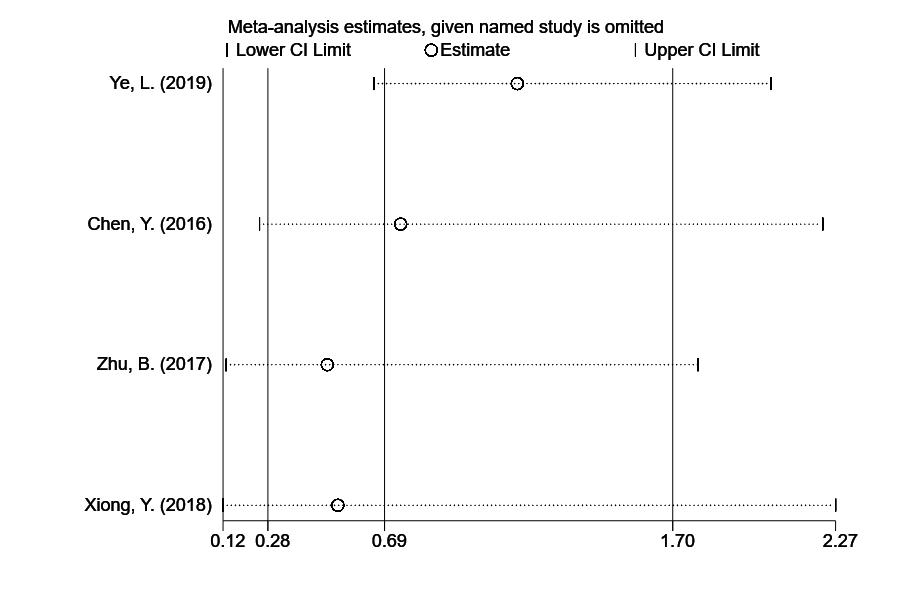
**

**G**


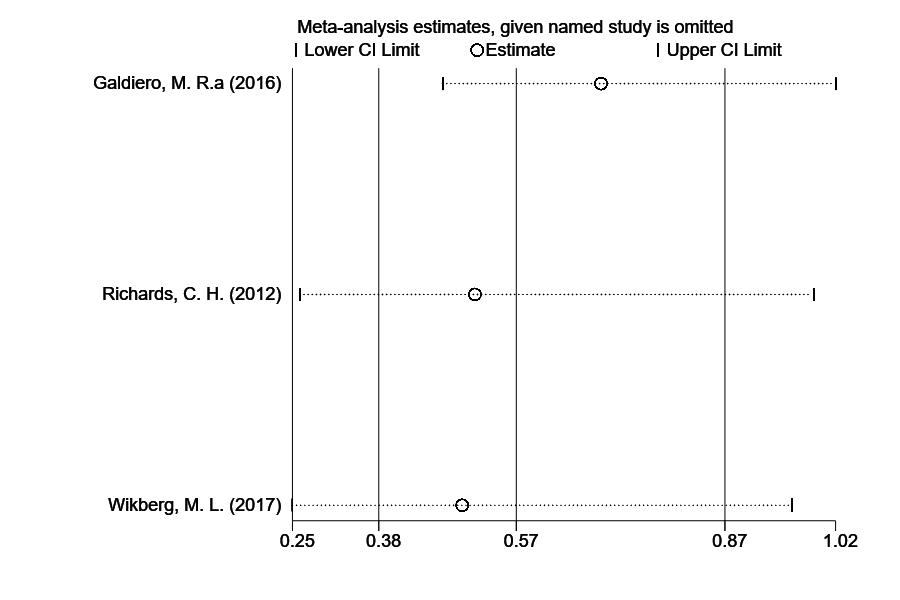


**Supplementary Figure 2.** Results of sensitivity analysis. (A-C) Sensitivity analysis of studies evaluated the expression of intratumoral, peritumoral and unclear compartments TANs on overall survival. (D-F) Sensitivity analysis of studies evaluated the expression of intratumoral, peritumoral and unclear compartments TANs on disease-free survival. (G) Sensitivity analysis of studies evaluated the expression of peritumoral TANs on cancer-specific survival.
